# Supplementary material for: SUMOylation controls the binding of hexokinase 2 to mitochondria and protects against prostate cancer tumorigenesis
Source: Nat Commun. 2021 Mar 22;12:1812. doi: 10.1038/s41467-021-22163-7 (PMC7985146; doi:10.1038/s41467-021-22163-7)
Supplement: Supplementary file 1 — Supplementary Information [file 41467_2021_22163_MOESM1_ESM.pdf]

SUMOylation Controls the Binding of Hexokinase 2 to Mitochondria and Protects against Prostate Cancer Tumorigenesis

Xun Shangguan, Jianli He, Zehua Ma, *et al.*

Supplementary Information

Contents

Supplementary Figure 1-7

Supplementary Table 1-5

a

Position	Group	JASSA	GPS-SUMO	R. Hay
K315	LILVKM**AKEE**LLFGG	*	*	**S,R**
K492	LEVKRR**MKVE**MERGL	*	***	**S,R**

b

HK	Peptide(308-322)	Peptide(485-499)
HK1	LILVKM**AKEG**LLFEG	LEVKKR**MRAE**MELGL
HK2	LILVKM**AKEE**LLFGG	LEVKRR**MKVE**MERGL

c

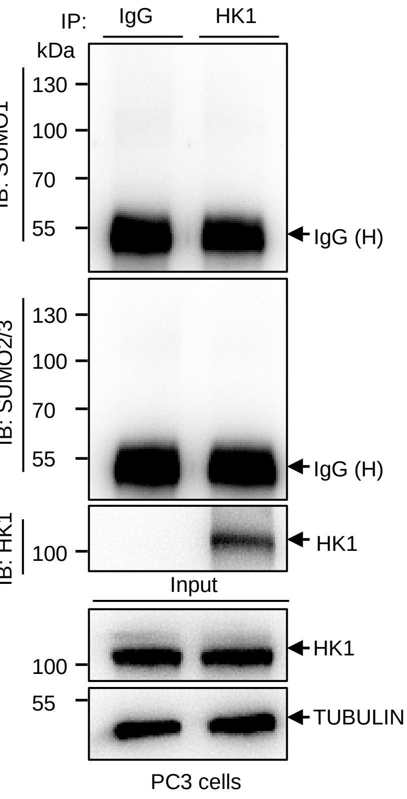

Supplementary Figure 1. HK1 is not the target of SUMOylation. (**a**) SUMO-sp2 predicted two potential SUMOylation site Lys(K) 315 and Lys(K) 492 for HK2. Analysis using R. Hay website provides motif type (S = strict and R= relaxed). (**b**) Sequence alignment of HK1 and HK2. The SUMO modification motif is highly conserved in HK2 but not presented in HK1. (**c**) Immunoprecipitation with IgG or anti-HK1 antibody and then Western blotting with anti-SUMO1 or anti-SUMO2/3 antibodies. No endogenous HK1 SUMOylation band was detected. Source data are provided as a Source Data file.

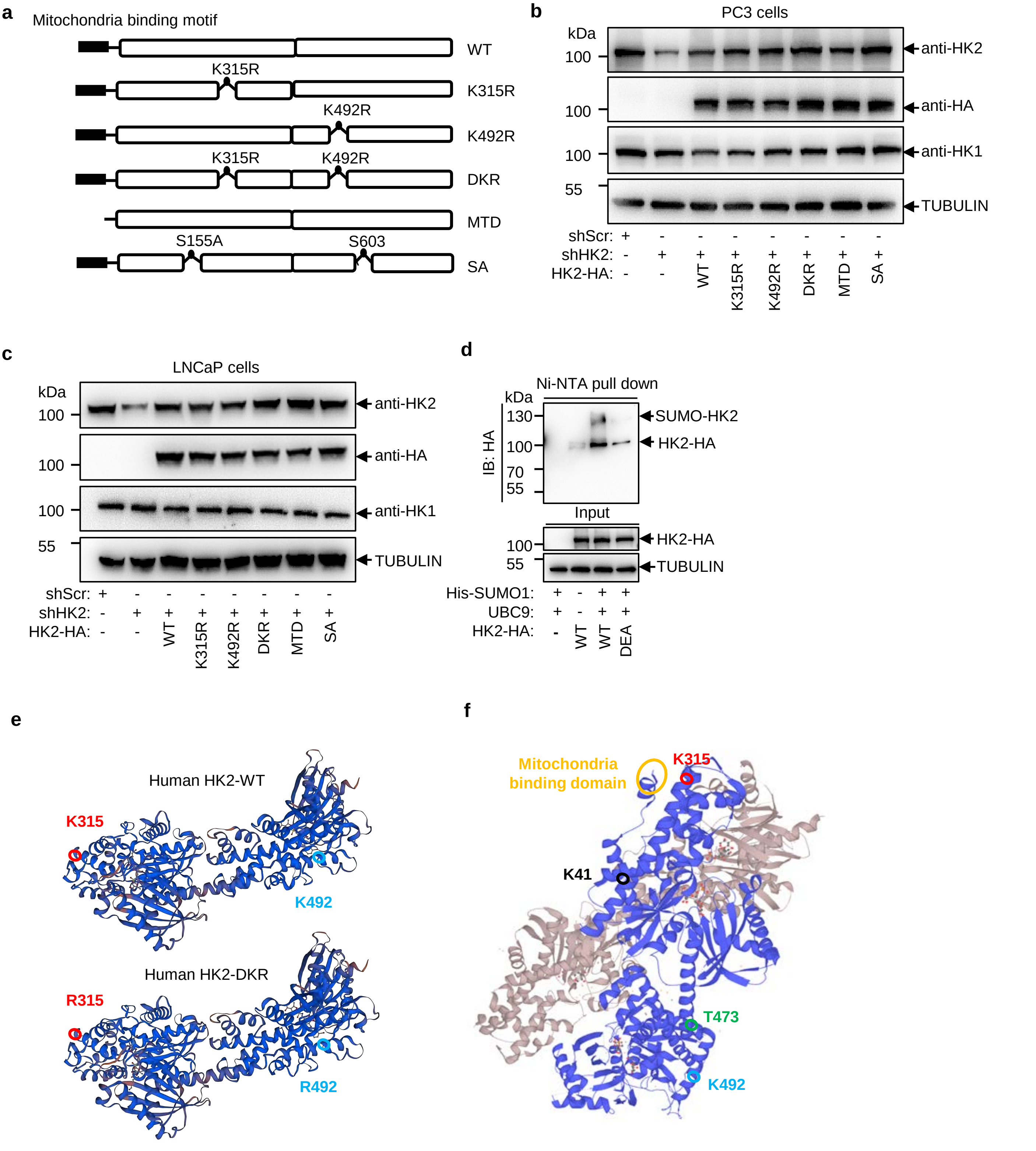

Supplementary Figure 2

Supplementary Figure 2. SUMO1 is conjugated to HK2 on Lys 315 and Lys 492. (**a**) Schematic diagram of wild-type HK2 and its mutant constructs used in the study. N terminal black bar represents the mitochondrial binding deficient mutant (MTD); SA: S155/603A-kinase dead mutant. (**b**) and (**c**) Western blotting for the indicated HK2 constructs from (a) and endogenous HK1 expression. (**d**) 293T cells were transfected with HA-tag *HK*2-WT or *HK2*-DEA with or without *His-SUMO1* and *UBC9*. Cell lysates were prepared for precipitation with Ni^{2+}-NTA resin, followed by Western blotting with indicated antibodies. No SUMOylation band was detected for the HK2 DEA mutant. (**e**) The human HK2-WT and HK2-DKR protein structure were generated using SWISS-MODEL (https://swissmodel.expasy.org/). (**f**) The human HK2 protein structure, generated at http://www.rcsb.org (PDB 2nzt). Source data are provided as a Source Data file.

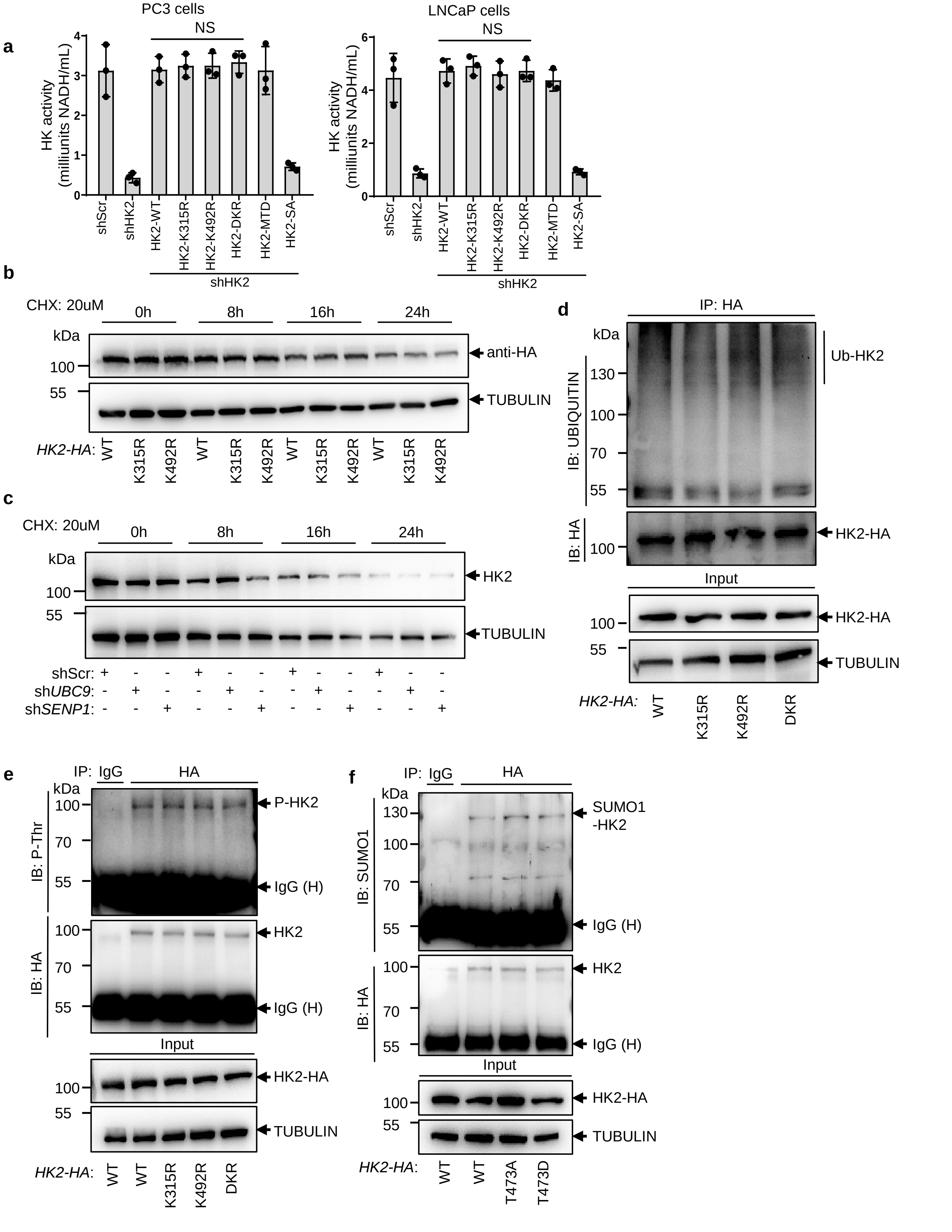

Supplementary Figure 3

Supplementary Figure 3. SUMOylation of HK2 does not influence its stability and hexokinase activity. (**a**) Hexokinase activity was measured in PC3 and LNCaP cells. Different mutant forms of *HK2* were stably expressed in cells, instead of endogenous *HK2*. In vitro hexokinase activity did not change in SUMO-defective HK2 cell line, compared with wild type. Data are presented as mean ± SEM of 3 biologically independent samples. Statistical significance was determined by two-tailed Student's t test. NS: not significant. (**b**) PC3 cells overexpressing different mutant forms of *HK2* were treated with protein synthesis inhibitor cycloheximide (CHX) at different time points. Western blotting showed the expression of HK2 protein. (**c**) PC3 cell knockdown *UBC9* or *SENP1* were treated with protein synthesis inhibitor CHX, and pulse-chased the HK2 protein. Western blotting showed the expression of HK2 protein. (**d**) SUMOylation of HK2 does not influence its ubiquitination. *HK2*-HA constructs (*HK2*-WT, *HK2*-K315R, *HK2*-K492R, *HK2*-DKR) were transfected into 293T cells. Ubiquitination was detected by immunoprecipitation with HA antibody and then Western blotting with ubiquitination antibody. (**e**) SUMOylation of HK2 does not influence its phosphorylation. *HK2-HA* constructs (*HK2*-WT, *HK2*-K315R, *HK2*-K492R) were transfected into 293T cells. Phosphorylation of HK2 was detected by immunoprecipitation with HA antibody and then Western blotting with Phospho-Threonine antibody. (**f**) Phosphorylation of HK2 does not influence its SUMOylation. *HK2-HA* constructs (*HK2*-WT, *HK2*-T473A, *HK2*-T473D) were transfected into 293T cells. SUMOylation of HK2 was detected by immunoprecipitation with HA antibody and then Western blotting with SUMO1 antibody. Source data are provided as a Source Data file.

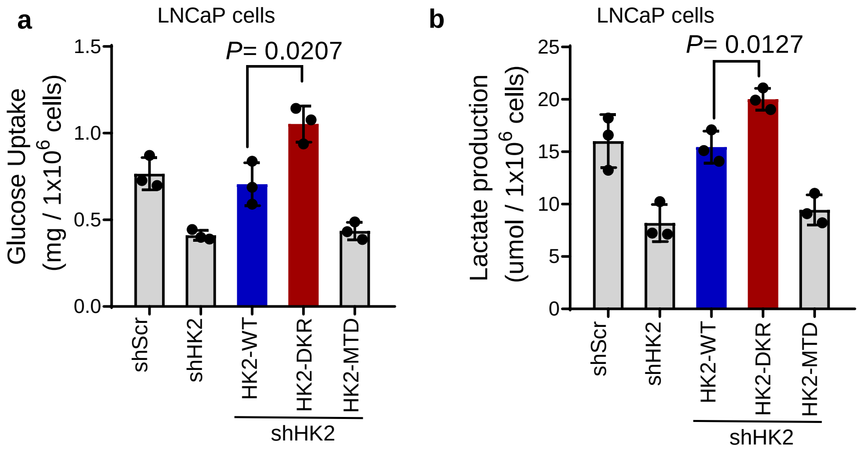

Supplementary Figure 4. SUMO-defective HK2 increases prostate cancer cell glycolysis. (**a**) Glucose consumption was measured in LNCaP cells with different mutant forms HK2. Endogenous *HK2* was knockdown by shRNA and replaced by different mutant forms with HA tag in PC3 cells. Data are presented as mean ± SEM of 3 biologically independent samples. Statistical significance was determined by two-tailed Student's t test. (**b**) Lactate production was measured in LNCaP cells with different mutant forms HK2. Data are presented as mean ± SEM of 3 biologically independent samples. Statistical significance was determined by two-tailed Student's t test. Source data are provided as a Source Data file.

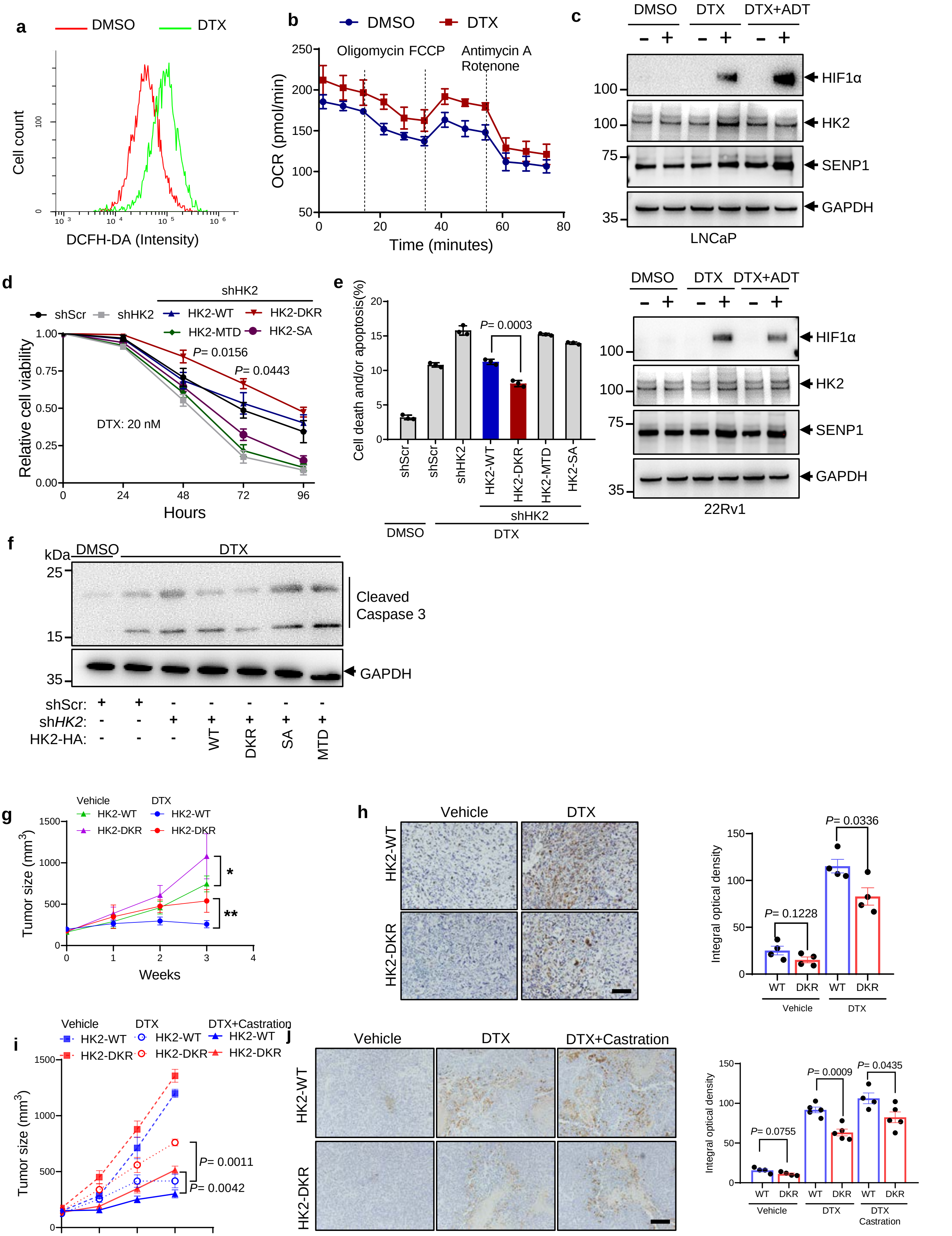

Supplementary Figure 5

Supplementary Figure 5. SUMO-defective HK2 conferred to worse chemotherapy response. (**a**) Docetaxel treatment elevated intracellular ROS level. Cells were treated with 20nM docetaxel or DMSO and measured by FACS. (**b**) PC3 cell with or without docetaxel treatment were measured for OCR with Seahorse metabolic analysis (*n*=3 independent experiments). (**c**) Western blotting analysis of HIF1α, HK2, and SENP1 expression in LNCaP and 22Rv1 cells with or without docetaxel/ADT treatment. (**d**) Cell viability were observed with docetaxel treatment at the indicated time points. PC3 stably expressed different mutant form of HK2 as indicated. Data are presented as mean ± SEM of 3 independent samples. Statistical significance was determined by two-tailed Student's t test. (**e**) PC3 cells with docetaxel treatment were measured for cell death and apoptosis with Annexin-V/PI staining. Data are presented as mean ± SEM of 3 independent samples. Statistical significance was determined by two-tailed Student's t test. (**f**) Western Blotting showed Cleaved Caspase-3 level in PC3 cells treated with docetaxel for 48 hrs. (**g**) PC3 cells were injected subcutaneously into male nude mice (n=4 mice per group). After tumors grew to about 150 mm^3 , mice were received intraperitoneal vehicle or docetaxel. Data are represented as means ± SEM. Statistical significance was determined by two-tailed Student's t test. (**h**) Representative images of Cleaved Caspase-3 staining of the PC3 tumors (*n*=4 samples per group) (left), and relative intensity was quantified by ImageJ (right). Scale bar, 200 μm. Data are represented as means ± SEM. Statistical significance was determined by two-tailed Student's t test. (**i**) 22Rv1 cells were injected subcutaneously into male nude mice (n=5 mice per group). After tumors grew to about 150 mm^3 , mice were received intraperitoneal vehicle or docetaxel, with or without castration. Data are represented as means ± SEM. Statistical significance was determined by two-tailed Student's t test. (**j**) Representative images of Cleaved Caspase-3 staining of the 22Rv1 tumors (*n*=5 samples per group) (left), and relative intensity was quantified by ImageJ (right). Scale bar, 200 μm. Data are represented as means ± SEM. Statistical significance was determined by two-tailed Student's t test. Source data are provided as a Source Data file.

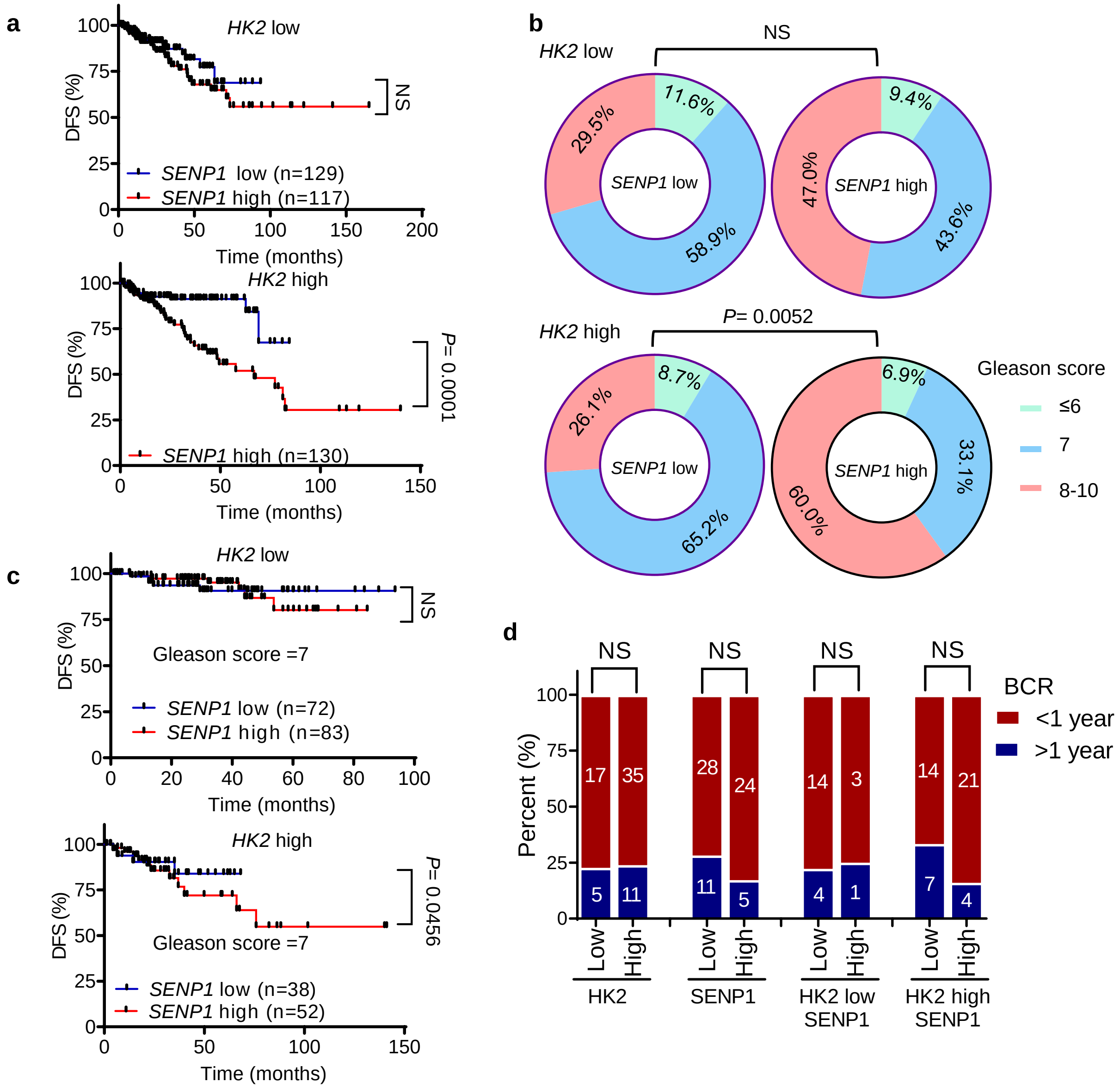

Supplementary Figure 6

Supplementary Figure 6. HK2 together with SENP1 upregulation associates poor outcome and worse chemotherapy response in prostate cancer patients. (**a**) Kaplan-Meier survival analysis in *HK2* high subgroup of prostate cancer disease-free survival (DFS) defined as *SENP1* low or high expression, using TCGA database (n= 491). Statistical difference was determined by two-sided log-rank test. NS: not significant. (**b**) Relationship between *SENP1* mRNA and Gleason Score in *HK2* low or high subgroup, using TCGA database. Gleason Score was divided in low ≤6, medium =7, high 8-10. The distribution of high Gleason Score increased in *SENP1* high group. Statistical significance was measured by Chi-square test. NS: not significant. (**c**) Kaplan-Meier survival analysis in Gleason score =7 subgroup (*n*=244 samples) of prostate cancer disease-free survival (DFS) defined as *SENP1* low or high expression, using TCGA database. Statistical difference was determined by two-sided log-rank test. NS: not significant. (**d**) The percentage of biochemical recurrence < 1 or ≥ 1 year of neoadjuvant treatment prostate cancer patients (*n*= 68), stratified by HK2 and SENP1 expression. Statistical significance was measured by Chi-square test. NS: not significant. Source data are provided as a Source Data file.

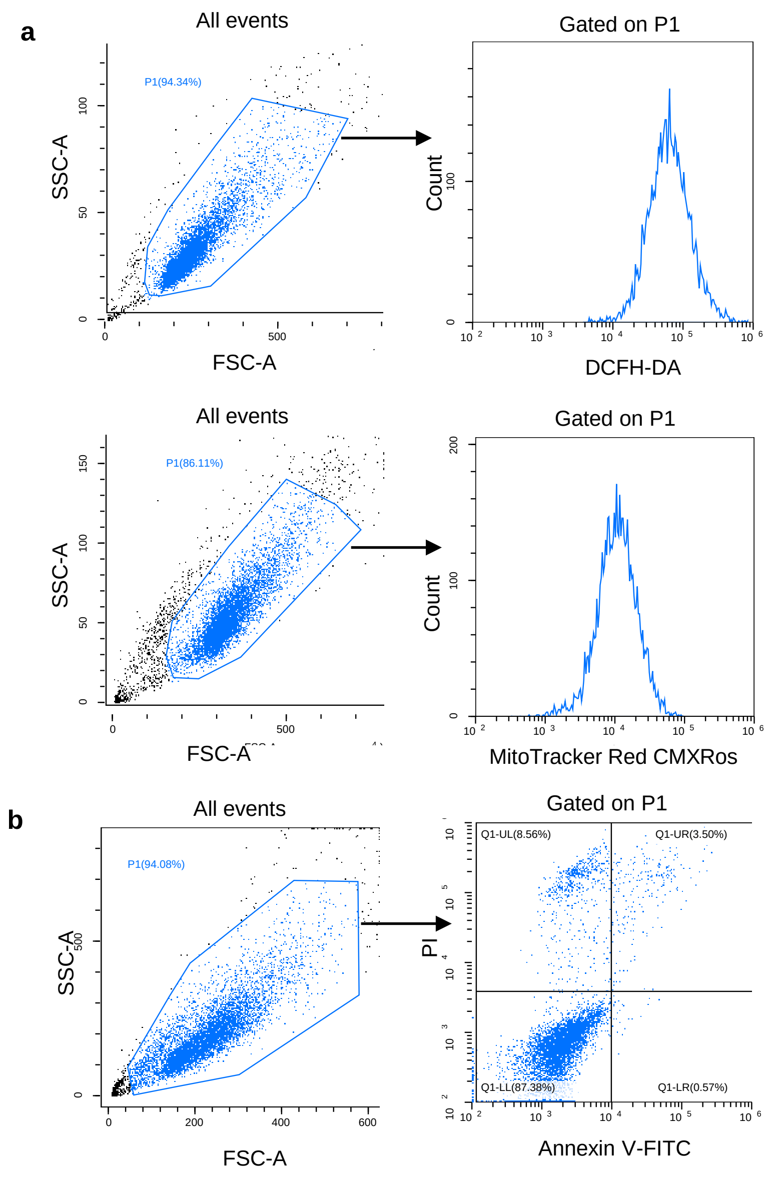

Supplementary Figure 7. Gating strategy for FACS. (**a**) Dot-plots and Histogram from FACS analysis to illustrate the gating to obtain the intensity of DCFH-DA or MitoTracker Red CMXRos staining in Figure 3a and S5a. (**b**) Dot-plots from FACS analysis to illustrate the gating to obtain the Annexin V^-PI^-, Annexin V^+PI^-, Annexin V^-PI^+, and Annexin V^+PI^+ in Figure S5e.

Supplementary Figure 7

Supplementary Table 1. shRNA sequences are listed.

sh*HK2*	5'-CCGGCCAAAGACATCTCAGACATTGCTCGAGCAATGTCTGAGATGTCTTTGGTTTTTG-3'
sh*UBC9*	5'-CCGGGCCTACACGATTTACTGCCAACTCGAGTTGGCAGTAAATCGTGTAGGCTTTTTG-3'
shScramble	5'-CCGGTTCCTGGAACAATTGCTTTTACTCGAGTAAAAGCAATTGTTCCAGGAATTTTTG-3'
sh*SENP1*	5'-CCGG AACTACATCTTCGTGTACCTC CTCGAGGAGGTACACGAAGATGTAGTT TTTTTG-3'
sh*SENP2*	5'-CCGG GCGAATTACTCGAGGAGATCTCGAGATCTCCTCGAGTAATTCGC TTTTTG-3'
sh*SENP3*	5'-CCGGGTGACCTGCGTACAGAGCACTCGAGTGCTCTGTACGCAGGTCAC TTTTTG-3'

Supplementary Table 2. Primers sequences are listed.

SENP1(Human)	Forward	AGTGAACCACAACTCCGTATTC
	Reverse	AAAAGATCGGTCCAAATGTCCTT
HK2(Human)	Forward	GAGCCACCACTCACCCTACT
	Reverse	CCAGGCATTCGGCAATGTG
HIF1A(Human)	Forward	GAACGTCGAAAAGAAAAGTCTCG
	Reverse	CCTTATCAAGATGCGAACTCACA
GAPDH(Human)	Forward	GGAGCGAGATCCCTCCAAAAT
	Reverse	GGCTGTTGTCATACTTCTCATGG

Supplementary Table 3. Summary of the clinical information of TCGA.

Variable	N (%) or median (IQR)
Subject	491
Age(years)	61（56-66）
Gleason score	
≤6	45（9.2%）
7	245（49.9%）
≥8	201（40.9%）
Tumor stage	
T2	185 (37.7%)
T3-T4	300 (61.1%)
Tx	6 (1.2%)
Metastasis stage	
M0	450 (91.7%)
M1	2 (0.4%)
Mx	39 (7.9%)
Recurrence	66（13.4%）
DFS (months)	27.07(14.03-45.11)
Follow-up (months)	30.55（17.21-48.09)

IQR interquartile range, DFS: Disease-free survival

Supplementary Table 4. The clinical information of prostate cancer cohort.

Variable	N (%) or median (IQR)
Subject	121
Age(years)	66（61-71）
Initial PSA（ng/ml）	16.05（10.48-35.14）
Gleason score	
≤6	26（20.5%）
7	48（39.7%）
≥8	47（38.8%）
Pathology stage	
T2	98 (81.0%)
T3-T4	23 (19.0%)
Adverse pathology	
ECE	5 (4.1%)
SVI	12 (9.9%)
PSM	6 (6.0%)
LI	4 (3.3%)
BPFS (months)	24.0 (15.1-30.4)
BCR	48（39.7%）
Year of surgery	2004-2013

IQR interquartile range, PSA prostate-specific antigen, ECE extracapsular extension, SVI seminal vesicle invasion, PSM positive surgical margins, LI lymph node invasion, BCR biochemical recurrence, BPFS biochemical progression free survival

Supplementary Table 5. The clinical information of patients received NHT or NCHT therapy prior to radical prostatectomy.

Variable	N (%) or median (IQR)	
	NHT group	NCHT group
Subject	68	48
Age(years)	68（65-72）	65（59.25-69.75）
Initial PSA（ng/mL）	70.55（48.00-117.29）	95.99（46.08-154.00）
Preoperative PSA（ng/mL）	0.68 (0.15-4.22)	0.42 (0.09-1.04)
Gleason score		
≤6	4（5.9%）	0（0%）
7	26（38.2%）	25（52.1%）
≥8	38（55.9%）	23（47.9%）
Clinical stage		
T2	30 (44.1%)	15 (31.2%)
T3	29 (42.7%)	20 (41.7%)
T4	9 (13.2%)	13 (27.1%)
Pathologic stage		
T2	36 (53.0%)	32 (66.7%)
T3	30 (44.1%)	13 (27.1%)
T4	2 (2.9%)	3 (6.2%)
Adverse pathology		
ECE	33 (48.5%)	12 (25.0%)
SVI	15 (22.1%)	11 (22.9%)
PSM	16 (23.5%)	8 (16.7%)
LI	13 (19.1%)	20 (41.7%)
BCR<1 year		
yes	52 (76.5%)	32 (66.7%)
no	16 (23.5%)	16 (33.3%)
Follow-up (months)	17.7（10.8-30.2)	13.3（9.1-18.6)
Year of surgery	2014-2017	2015-2017

IQR interquartile range, PSA prostate-specific antigen, ECE extracapsular extension, SVI seminal vesicle invasion, PSM positive surgical margins, LI lymph node invasion, BCR biochemical recurrence, NHT neoadjuvant hormonal therapy, NCHT neoadjuvant chemohormonal therapy.
