# Supplementary material for: SUMOylation controls the binding of hexokinase 2 to mitochondria and protects against prostate cancer tumorigenesis
Source: Nat Commun. 2021 Mar 22;12:1812. doi: 10.1038/s41467-021-22163-7 (PMC7985146; doi:10.1038/s41467-021-22163-7)
Supplement: Supplementary file 3 — Reporting Summary [file 41467_2021_22163_MOESM3_ESM.pdf]

nature research

Corresponding author(s): Qi Wang, Wei Xue, Jinke Cheng

Last updated by author(s): Jan 6, 2021

Reporting Summary

Nature Research wishes to improve the reproducibility of the work that we publish. This form provides structure for consistency and transparency in reporting. For further information on Nature Research policies, see Authors & Referees and the Editorial Policy Checklist .

Statistics

For all statistical analyses, confirm that the following items are present in the figure legend, table legend, main text, or Methods section.

n/a	Confirmed	
☐	☒	The exact sample size (*n*) for each experimental group/condition, given as a discrete number and unit of measurement
☐	☒	A statement on whether measurements were taken from distinct samples or whether the same sample was measured repeatedly
☐	☒	The statistical test(s) used AND whether they are one- or two-sided<br>*Only common tests should be described solely by name; describe more complex techniques in the Methods section.*
☒	☐	A description of all covariates tested
☒	☐	A description of any assumptions or corrections, such as tests of normality and adjustment for multiple comparisons
☐	☒	A full description of the statistical parameters including central tendency (e.g. means) or other basic estimates (e.g. regression coefficient) AND variation (e.g. standard deviation) or associated estimates of uncertainty (e.g. confidence intervals)
☐	☒	For null hypothesis testing, the test statistic (e.g. *F*, *t*, *r*) with confidence intervals, effect sizes, degrees of freedom and *P* value noted<br>*Give P values as exact values whenever suitable.*
☒	☐	For Bayesian analysis, information on the choice of priors and Markov chain Monte Carlo settings
☒	☐	For hierarchical and complex designs, identification of the appropriate level for tests and full reporting of outcomes
☐	☒	Estimates of effect sizes (e.g. Cohen's *d*, Pearson's *r*), indicating how they were calculated

Our web collection on statistics for biologists contains articles on many of the points above.

Software and code

Policy information about availability of computer code

Data collection	None computer code
Data analysis	ImageJ v. 1.45, Graphpad PRISM8, GSEA 4.1.0, CytExpert 2.0, R 4.0.2.

For manuscripts utilizing custom algorithms or software that are central to the research but not yet described in published literature, software must be made available to editors/reviewers. We strongly encourage code deposition in a community repository (e.g. GitHub). See the Nature Research guidelines for submitting code & software for further information.

Data

Policy information about availability of data

All manuscripts must include a data availability statement. This statement should provide the following information, where applicable:

- Accession codes, unique identifiers, or web links for publicly available datasets
- A list of figures that have associated raw data
- A description of any restrictions on data availability

The gene expression profile of GSE83654, GSE33455, and GSE36135 were obtained from the National Center for Biotechnology Information (NCBI) Genome database (https://www.ncbi.nlm.nih.gov). The Cancer Genome Atlas (TCGA) human prostate cancer data set (TCGA Firehose Legacy) were downloaded from cBioPortal for Cancer Genomics (http://cbioportal.org). All other data supporting the findings of this study are available from the corresponding author upon reasonable request. A Reporting Summary for this study is available as a Supplementary Information file. Source data are provided with this paper.

Field-specific reporting

Please select the one below that is the best fit for your research. If you are not sure, read the appropriate sections before making your selection.

Life sciences study design

All studies must disclose on these points even when the disclosure is negative.

Sample size	We aimed for at least 4 animals per group to allowed basic statistical analysis, the sample size was different in each experiment(4-8), which gave sufficient statistics and supported meaningful conclusions. The exact number of mice was indicated in figure legends. For in vitro experiment, each experiment was repeated at least 3 times to confirm reproducibility.
Data exclusions	Mice that died for unknown reasons were excluded from analysis.
Replication	For in vitro experiment, each experiment was repeated at least 3 times with similar results. For animal experiment at least 4 mice were used in each group. All findings were replicated successfully.
Randomization	Mice were randomly selected for tumor implantation and randomly grouped for docetaxel or vehicle treatment. Where possible, animals used in one experiment were age-matched.
Blinding	For animal experiments, animals of indicated group were selected and assigned randomly, data collection and analysis for IHC were performed blindly. Humane endpoint monitoring of all mice of all groups was carried out by the same member of staff at the animal facility according to pre-defined and approved methods (as defined by Shanghai Jiao Tong University School of Medcine Animal Care Committee). This ensured consistent treatment of all groups.

Reporting for specific materials, systems and methods

We require information from authors about some types of materials, experimental systems and methods used in many studies. Here, indicate whether each material, system or method listed is relevant to your study. If you are not sure if a list item applies to your research, read the appropriate section before selecting a response.

Materials & experimental systems

n/a	Involved in the study	
☐	☒	Antibodies
☐	☒	Eukaryotic cell lines
☒	☐	Palaeontology
☐	☒	Animals and other organisms
☐	☒	Human research participants
☒	☐	Clinical data

Methods

n/a	Involved in the study	
☒	☐	ChIP-seq
☐	☒	Flow cytometry
☒	☐	MRI-based neuroimaging

Antibodies

Antibodies used

1. anti-SENP1 Abcam ab108981
2. anti-SENP2 Abcam ab58418
3. anti-HIF-1α Cell Signaling Technology 79233
4. anti-Phospho-Threonine Cell Signaling Technology 9386
5. anti-Ubc9 Cell Signaling Technology 4786
6. anti-BrdU Cell Signaling Technology 5292
7. anti-Ubiquitin Cell Signaling Technology 3936
8. anti-Cleaved-caspase 3 Cell Signaling Technology 9661
9. anti-SUMO1 Cell Signaling Technology 4930
10. anti-SUMO2/3 Cell Signaling Technology 4971
11. anti-SENP1 Cell Signaling Technology 11929
12. anti-SENP3 Cell Signaling Technology 5591
13. anti-HA-Tag Cell Signaling Technology 3724
14. anti-Flag-Tag Cell Signaling Technology 14793
15. anti-Hexokinase 1 Proteintech 19662-1-AP
16. anti-Hexokinase 2 Proteintech 22029-1-AP
17. anti-VDAC1 Proteintech 10866-1-AP
18. anti-alpha Tubulin Proteintech 66031-1-Ig
19. anti-GAPDH Proteintech 10494-1-AP
20. anti-Rabbit IgG H&L (Alexa Fluor® 488) Abcam ab150061

Validation

All antibodies have been validated on the product webpages or literature, including:
1. anti-SENP1 antibody has been validated for WB and IHC of human samples by previous publications (e.g .PMID: 32232156, PMID: 32104241, and PMID: 31186231). https://www.abcam.cn/senp1-antibody-epr3844-ab108981.html
2. anti-SENP2 antibody has been validated for WB of human samples by previous publications (e.g .PMID: 32290845 and PMID:

20587419). https://www.abcam.cn/senp2-antibody-ab58418.html
3. anti-HIF-1α antibody has been validated for WB of human samples by previous publications (e.g .PMID: 31591207 and PMID: 31175094). https://www.cellsignal.cn/products/primary-antibodies/hif-1a-d5f3m-mouse-mab/79233?site-search-type=Products&N=4294956287&Ntt=79233&fromPage=plp&_requestid=1479104
4. anti-Phospho-Threonine antibody has been validated for WB of human samples by previous publications (e.g .PMID: 27555448, PMID: 31578312, and PMID: 31537781). https://www.cellsignal.cn/products/primary-antibodies/phospho-threonine-42h4-mouse-mab/9386?site-search-type=Products&N=4294956287&Ntt=9386&fromPage=plp&_requestid=1479186
5. anti-Ubc9 antibody has been validated for WB of human samples by previous publications (e.g .PMID: 26074333 and PMID: 26659182). https://www.cellsignal.cn/products/primary-antibodies/ubc9-d26f2-xp-rabbit-mab/4786?site-search-type=Products&N=4294956287&Ntt=4786&fromPage=plp&_requestid=1479206
6. anti-BrdU antibody has been validated for IF of human samples by previous publications (e.g .PMID: 31949145, PMID: 31431624, and PMID: 31257532). https://www.cellsignal.cn/products/primary-antibodies/brdu-bu20a-mouse-mab/5292?site-search-type=Products&N=4294956287&Ntt=5292&fromPage=plp&_requestid=1479233
7. anti-Ubiquitin antibody has been validated for WB of human samples by previous publications (e.g .PMID: 32472079, PMID: 32461552, and PMID: 32404918). https://www.cellsignal.cn/products/primary-antibodies/ubiquitin-p4d1-mouse-mab/3936?site-search-type=Products&N=4294956287&Ntt=3936&fromPage=plp&_requestid=1479255
8. anti-Cleaved-caspase 3 antibody has been validated for WB and IHC of human samples by previous publications (e.g .PMID: 32934208, PMID: 32894152, and PMID: 32705208). https://www.cellsignal.cn/products/primary-antibodies/cleaved-caspase-3-asp175-antibody/9661?site-search-type=Products&N=4294956287&Ntt=9661&fromPage=plp&_requestid=1479272
9. anti-SUMO1 antibody has been validated for WB and IP of human samples by previous publications (e.g .PMID: 31932588 and PMID: 31575873). https://www.cellsignal.cn/products/primary-antibodies/sumo-1-antibody/4930?site-search-type=Products&N=4294956287&Ntt=4930&fromPage=plp&_requestid=1479289
10. anti-SUMO2/3 antibody has been validated for WB and IP of human samples by previous publications (e.g .PMID: 32641734, PMID: 31575873, and PMID: 30472188). https://www.cellsignal.cn/products/primary-antibodies/sumo-2-3-18h8-rabbit-mab/4971?site-search-type=Products&N=4294956287&Ntt=4971&fromPage=plp&_requestid=1479347
11. anti-SENP1 antibody has been validated for WB of human samples by previous publications (e.g .PMID: 2884255 and PMID: 28947497). https://www.cellsignal.cn/products/primary-antibodies/senp1-d16d7-rabbit-mab/11929?site-search-type=Products&N=4294956287&Ntt=11929&fromPage=plp&_requestid=1479379
12. anti-SENP3 antibody has been validated for WB of human samples by previous publications (e.g .PMID: 29352108 and PMID: 31141694). https://www.cellsignal.cn/products/primary-antibodies/senp3-d20a10-xp-rabbit-mab/5591?site-search-type=Products&N=4294956287&Ntt=5591&fromPage=plp&_requestid=1479411
13. anti-HA-Tag antibody has been validated for WB, IP, and IF of human samples by previous publications (e.g .PMID: 32866139, PMID: 32641734, and PMID: 32172343). https://www.cellsignal.cn/products/primary-antibodies/ha-tag-c29f4-rabbit-mab/3724?site-search-type=Products&N=4294956287&Ntt=3724&fromPage=plp&_requestid=1479434
14. anti-Flag-Tag antibody has been validated for WB, IP, and IF of human samples by previous publications (e.g .PMID: 32413996, PMID: 32182354, and PMID: 32366851). https://www.cellsignal.cn/products/primary-antibodies/dykddddk-tag-d6w5b-rabbit-mab-binds-to-same-epitope-as-sigma-s-anti-flag-m2-antibody/14793?site-search-type=Products&N=4294956287&Ntt=14793&fromPage=plp&_requestid=1479460
15. anti-Hexokinase 1 antibody has been validated for WB of human samples by previous publications (e.g .PMID:30944034, PMID:27382509, and PMID: 30515768). http://www.ptgcn.com/products/HK1-Antibody-19662-1-AP.htm
16. anti-Hexokinase 2 antibody has been validated for WB, IP, IHC, and IF of human samples by previous publications (e.g .PMID: 30719175, PMID: 30825877, and PMID: 30723389). http://www.ptgcn.com/products/HK2-Antibody-22029-1-AP.htm
17. anti-VDAC1 antibody has been validated for WB of human samples by previous publications (e.g .PMID: 30555042, PMID: 30916346, and PMID: 30952814). http://www.ptgcn.com/products/VDAC1-Antibody-10866-1-AP.htm
18. anti-alpha Tubulin antibody has been validated for WB of human samples by previous publications (e.g .PMID: 30733336, PMID: 30695697, and PMID: 30917319). http://www.ptgcn.com/products/tubulin-Alpha-Antibody-66031-1-Ig.htm
19. anti-GAPDH antibody has been validated for WB of human samples by previous publications (e.g .PMID: 30523220, PMID: 30555157, and PMID: 30613277). http://www.ptgcn.com/products/GAPDH-Antibody-10494-1-AP.htm
20. anti-Rabbit IgG H&L (Alexa Fluor® 488) antibody has been validated for IF by previous publications (e.g .PMID: 30837464, PMID: 30836676, and PMID: 31704931). https://www.abcam.cn/donkey-rabbit-igg-hl-alexa-fluor-488-preadsorbed-ab150061.html

Eukaryotic cell lines

Policy information about cell lines

Cell line source(s)	LNCaP, 22Rv1, PC3, and 293T cells were purchased from the Cell Bank, Shanghai Institutes for Biological Sciences, Chinese Academy of Sciences.
Authentication	All cell lines were authenticated by autosomal STR profiling.
Mycoplasma contamination	All the cell lines were tested negative for mycoplasma contamination.
Commonly misidentified lines (See ICLAC register)	None of commonly misidentified cell lines were used.

Animals and other organisms

Policy information about studies involving animals; ARRIVE guidelines recommended for reporting animal research

Laboratory animals	5-7 weeks old BALB/c nude male mice were used.
Wild animals	No wild animals were used.
Field-collected samples	No field-collected samples were used.
Ethics oversight	All animals were maintained in a pathogen-free environment. Animal experiments were conducted according to the protocols approved by the Animal Care Committee of Shanghai Jiao Tong University School of Medicine.

Note that full information on the approval of the study protocol must also be provided in the manuscript.

Human research participants

Policy information about studies involving human research participants

Population characteristics	All patients were diagnosed with prostate cancer. All tumors specimens were collected at the time of radical prostatectomy. For prostate cancer cohort, their characteristics were: median age: 66 years, median Initial PSA: 16.05 ng/mL, 100% male, median BPFS: 24.0 months, 39.7% BCR. For NHT cohort, their characteristics were: median age: 68 years, median Initial PSA: 70.55 ng/mL, 100% male, median follow-up time : 17.7 months, BCR<1 year: 76.5%. For NCHT cohort, their characteristics were: median age: 65 years, median Initial PSA: 95.99 ng/mL, 100% male, median follow-up time : 13.3 months, BCR<1 year: 66.7%.
Recruitment	All patients were approached without bias toward baseline characteristics such as, but not limited to, age, ethnicity, genetic/familial predisposition for cancer, tumor stage, tumor grade, or histology. Patients may have declined participation for personal reasons, which were independent of the study team. Accordingly, patient selection was unlikely to impact results.
Ethics oversight	All studies with human research participants were approved by the Shanghai Jiao Tong University School of Medicine, Renji Hospital Ethics Committee.

Note that full information on the approval of the study protocol must also be provided in the manuscript.

Flow Cytometry

Plots

Confirm that:

- [x] The axis labels state the marker and fluorochrome used (e.g. CD4-FITC).
- [x] The axis scales are clearly visible. Include numbers along axes only for bottom left plot of group (a 'group' is an analysis of identical markers).
- [x] All plots are contour plots with outliers or pseudocolor plots.
- [x] A numerical value for number of cells or percentage (with statistics) is provided.

Methodology

Sample preparation	For cell apoptosis and cell death assay, PC3 cells were seeded in 6 well plates. The next day, fresh media containing Docetaxel (20 nM), or control (0.1% DMSO) were added and cells incubated for two days. After treatment, the cells were trypsinized, collected, and stained with annexin V-fluorescein isothiocyanate (FITC) and propidium iodide (PI) simultaneously using an Annexin V-FITC Apoptosis Detection kit (Invitrogen, V13242). The cell suspensions were analyzed with a Beckman Coulter flow cytometer to determine the percentage of apoptotic (FITC stained cells) and necrotic cells (PI stained cells).<br>In addiction, ROS levels was determined by using DCFH (DCFH-DA, Sigma, D6883). Briefly, cells with specified treatments were washed with PBS and incubated with 10 μM DCFH-DA at 37°C for 30 min to load the fluorescent dye. Afterward, cells were washed twice with PBS and trypsinized for ROS detection by FACS
Instrument	BECKMAN COULTER DxFLEX
Software	CytExpert 2.0
Cell population abundance	Cells were analyzed without sorting.
Gating strategy	Cells were first gated by SSC and FSC to remove cell debris. Cell death or ROS level was quantified using CytExpert 2.0.

- [x] Tick this box to confirm that a figure exemplifying the gating strategy is provided in the Supplementary Information.
